# Formulation Development and Release Studies of Indomethacin Suppositories

**DOI:** 10.4103/0250-474X.44602

**Published:** 2008

**Authors:** M. L. Sah, T. R. Saini

**Affiliations:** Department of Pharmacy, Shri Govindram Seksaria Institute of Technology and Science, 23, Park Road, Indore-452 003, India

**Keywords:** Indomethacin, *in vitro* evaluation, modified continuous flow through bead bed apparatus, PEG, suppositories

## Abstract

Indomethacin suppositories were prepared by using water-soluble and oil soluble suppository bases, and evaluated for *in vitro* release by USP I and modified continuous flow through bead bed apparatus. Effect of the Tween 80 (1% and 5%) was further studied on *in vitro* release of the medicament. Release rate was good in water-soluble suppositories bases in comparison to oil soluble suppositories bases. Release was found to be greater in modified continuous flow through bead bed apparatus. When surfactant was used in low concentration then release rate was much greater, as compared to high concentration. When stability studies were performed on the prepared indomethacin suppositories it was found that suppositories made by water-soluble base had no significant changes while suppositories prepared by oil soluble bases, had some signs of instability.

Indomethacin, 1-(4-chlorobenzoyl)-5-methoxy-2-methylindol-3-yl-acetic acid^1^ a potent nonsteroidal antiinflammatory agent (NSAIA), has been used effectively in the management of moderate to severe rheumatoid arthritis, ankylosing spondilytis, osteoarthritis and acute gouty arthritis^2^–^4^. Like other NSAIAs, indomethacin causes irritation, nausea, anorexia, gastric bleeding and diarrhea when given orally^5^. Consequently, an alternate route of administration to avoid or minimize the above side effects is preferred in form of suppositories.

Indomethacin suppositories were formulated with oil soluble and water soluble suppository bases, using different combinations. *In vitro* release of the prepared suppositories were evaluated by USP I and modified continuous flow through bead bed cell. The effect of surfactant on *in vitro* release was also studied. Prepared suppositories were further kept for freeze-thaw and accelerated temperature conditions to study the stability of the prepared formulations.

Indomethacin was purchased from Alkem Laboratories Limited, Mumbai and polyethylene glycols were purchased from S. D. Fine Chemicals Ltd., Boisar. Mayol and Hydrokote AP-5 were procured from M/S Subhash Chemicals Industries, Pune. All other chemicals were of the analytical grade and used as procured.

The displacement values for all suppository bases (Table 1) were first determined^6^. Twelve formulae were devised using water-soluble and oil soluble suppository bases. Tween-80 was incorporated in the formulation to enhance the release of the drug from the formulation^7^. Out of twelve suppositories best seven suppositories were selected for further studies on the basis of physical characteristics. Suppositories were made by the moulding method^8^. Accurately weighed amount of the respective bases were melted on the water bath and maintained at 55°. The finely divided drug powder was then added to the melted mass and thoroughly mixed. The melt was then poured into the 1 g suppositories moulds and set aside for cooling for 15 min. The suppositories formed were taken out from the moulds and stored in refrigerator.

**TABLE 1:** COMPOSITION OF INDOMETHACIN SUPPOSITORIES

Formulation Code*	Excipients						
	
	PEG 400	PEG 1450	PEG 4000	PEG 6000	Tween-80	Mayol W-45	Hydrokote AP-5
WSIS-1	30%	41%	29%	-	-	-	-
WSIS-2	30%	41%	28%	-	1%	-	-
WSIS-3	29%	39%	27%	-	5%	-	-
WSIS-4	30%	40%	20%	10%	-	-	-
WSIS-5	70%	-	-	30%	-	-	-
WSIS-6	-	75%	-	25%	-	-	-
WSIS-7	60%	40%	-	-	-	-	-
WSIS-8	-	45%	-	55%	-	-	-
WSIS-9	-	50%	-	50%	-	-	-
WSIS-10	30%	70%	-	-	-	-	-
OSIS-11	-	-	-	-	-	100%	-
OSIS-12	-	-	-	-	-	-	100%

* WSIS: Water soluble indomethacin suppositories

* OSIS: Oil soluble indomethacin suppositories

Prepared suppositories were evaluated for release characteristics from the moulds, visual appearances, melting of suppositories in palm, stickiness, colour, brittleness and the hardness when pressed between thumb and index finger. All the suppositories (made by the respective bases and selected for further studies), were weighed and average weight was calculated. Then all the suppositories were individually weighed and the variation from the average was calculated (Table 2).

**TABLE 2:** EVALUATION OF DIFFERENT PARAMETERS OF SUPPOSITORIES

Formulation Code	Weight variation mg±SD	Drug content %±SD	Liquefaction time min ±SD	Breaking strength g±SD	Disintegration time min±SD
WSIS-1	850.40±0.012	99.03±0.66	7.45±0.01	500±0.23	4.08±0.047
WSIS-2	850.00±0.015	98.97±0.74	8.00±0.01	450±0.22	5.07±0.078
WSIS-3	848.09±0.015	99.56±0.88	7.30±0.02	500±0.18	5.00±0.034
WSIS-5	860.00±0.016	99.65±0.12	9.00±0.01	500±0.24	4.22±0.084
WSIS-6	852.45±0.014	98.89±0.15	11.00±0.01	450±0.19	5.39±0.057
OSIS-11	720.00±0.065	98.05±0.77	1.30±0.02	250±0.29	2.40±0.045
OSIS-12	700.00±0.017	97.99±0.89	1.00±0.21	150±0.31	2.14±0.076

Indomethacin, practically insoluble in water, is soluble in equal mixture of phosphate buffer pH 7.2 and methanol^9^. Three randomly selected suppositories were taken in 1000 ml standard flask containing 100 ml mixture of phosphate buffer pH 7.2 and methanol (50:50).The flask was shaken for desired period of time to dissolve the drug from suppositories. Absorbance of the resulting solutions after appropriate dilutions was measured on Shimadzu 160 A double beam UV/Vis spectrophotometer at 320 nm against the blank prepared using respective suppositories without drug (Table 2).

Liquefaction time of the suppositories was determined by modified Krowczynski method^10^, which is complementary to the determination of melting point. The apparatus measures the time necessary for a suppository to liquefy under pressure similar to those found in the rectum in the presence of water at body temperature. A glass tube with a stricture was filled with distilled water to adjust below the mark of stricture and heated in water bath to a temperature of 37±0.5°. A suppository was introduced in the tube and carefully pushed down its length until it sets on the top of stricture with the help of glass rod. The glass rod was continued to rest of the suppository till it reached the stricture due to the melting of suppository. The time taken by the glass rod to reach the stricture was determined as the liquefaction time of the suppository (Table 2).

Breaking strength of the suppository was determined with the help of apparatus, fabricated in the laboratory^11^. A suppository was introduced into the tube, having a stricture at its lower end, and a glass rod of length 120 mm was placed on the suppository. After that 200 g weight was put on the glass-rod and further weights were added at an interval of 1 min until the suppository collapsed. The weight required to break the suppository was calculated as follows: a) when suppository collapsed within 20 s of placing last weight then weight was not taken into account b) when suppository collapsed between 20 s and 40 s of placing the last weight then only half of the last weight was taken into calculation and c) when suppository remain uncrushed for more than 40 s after placing the last weight then all weights were used in the calculation (Table 2).

Disintegration time of the suppositories was determined on Electrodes disintegration tester USPED 2L. Six suppositories were weighed individually and placed in the tubes, which were then immersed in the beaker containing 900 ml of phosphate buffer pH 6.8 and instrument was run for 90 min at 37±0.5° (Table 2).

Suppositories were wrapped in the aluminum foil and kept in stressed condition by six cycles of freeze (2-8°) and thaw (25°) process. Suppositories were also kept in accelerated condition temperature (30°) for 45 days. Suppositories were examined visually and drug content was determined on a Shimadzu 160 A double beam UV/Vis spectrophotometer at 320 nm and results are shown in Table 3.

**TABLE 3:** STABILITY STUDIES OF INDOMETHACIN SUPPOSITORIES

Formulation	Freeze and thaw (six cycles)		Accelerated temperature (30°)	
		
code	Physical changes	% drug content±S.D.	Physical changes	% drug content±S.D.
WSIS-1	No significant changes were seen	98.93±0.12	No significant changes were seen	98.03±0.15
WSIS-2	No significant changes were seen	98.69±0.23	No significant changes were seen	98.06±0.34
WSIS-3	No significant changes were seen	99.35±0.43	No significant changes were seen	99.00±0.56
WSIS-5	No significant changes were seen	99.11±0.31	No significant changes were seen	98.19±0.71
WSIS-6	No significant changes were seen	97.96±0.35	No significant changes were seen	97.35±0.53
OSIS-11	Suppositories became harder	98.00±0.53	Suppositories became harder	98.01±0.75
OSIS12	Suppositories became too harder	97.09±0.06	Suppositories became too soft	97.01±0.04

*In vitro* release study was performed by using USP type1 rotating basket apparatus (Electrolab TDP-06P) and modified continuous flow through bead-bed apparatus (fabricated in the laboratory^11^). In the first method, dissolution medium was 500 ml mixed phosphate buffer pH 7.8. Rotation speed was controlled at 120 rpm while temperature was maintained at 37±0.5°. In the latter method, release studies were performed by using three rows of glass beads (chemical resistant, 3.5-4.5 mm in diameter) placed in release chamber. The suppository was inserted into the centre of the chamber using stainless steel forceps. The remaining glass beads were poured over the suppository. The mixed phosphate buffer (pH 7.8) was used as the dissolution medium, added in the reservoir and the peristaltic pump was started in the reverse direction allowing the fluid to fill the release chamber from the bottom. As soon as the entire release chamber was filled with dissolution medium the flow was reversed so that the liquid moves from the top to bottom of the chamber. The reservoir was insulated with polystyrene and stirred using magnetic stirrer. Dissolution fluid was maintained at temperature of 37±0.5° and flow rate of the dissolution medium was maintained at 16 ml/min. Five milliliter aliquots of the dissolution fluid were withdrawn at specified interval from the reservoir and each time replaced with equal volume of fresh dissolution medium. Withdrawn samples were suitably diluted and analyzed using Shimadzu 160A double beam UV/Vis spectrophotometer at 320 nm. All measurements were done in triplicate (figs. 1 and 2).

**Fig. 1:**
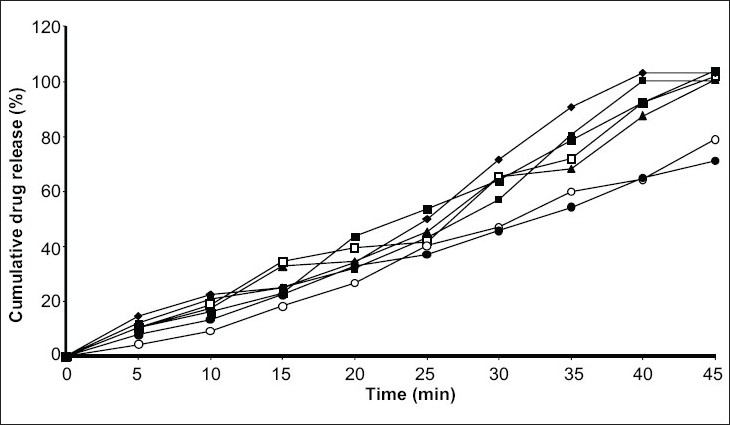
*In vitro* release of indomethacin suppositories by rotating basket method Release profiles of indomethacin suppositories from different water soluble bases-WSIS-1 (▲), WSIS-2 (▪), WSIS-3 (□), WSIS-5 (♦), WSIS-6 (■), and oil soluble bases OSIS-11 (◦), OSIS-12 (•)

**Fig. 2:**
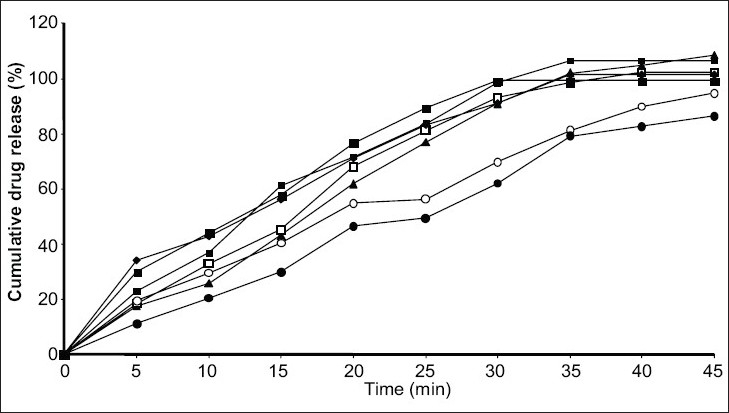
*In vitro* release profile of indomethacin suppositories by modified flow through cell apparatus Release profiles of indomethacin suppositories from different water soluble bases-WSIS-1 (▲), WSIS-2 (▪), WSIS-3 (□), WSIS-5 (♦), WSIS-6 (■), and oil soluble bases OSIS-11 (◦), OSIS-12 (•)

Suppositories were formulated by using water-soluble and oil soluble bases. Suppository made by water-soluble bases were yellow in colour, had good physical appearance and good mould release characteristics, whereas oil soluble suppositories were off white in colour. They were soft and had good mould release characteristics.

Hardness of the water-soluble bases and oil soluble bases were in the range of 650-700 g and 150-200 g respectively. Liquefaction time of the water soluble and oil soluble suppositories were 10-13 min and 1.30-2.00 min. Disintegration time of the water soluble and oil soluble suppositories were 4.30-5.40 minutes and 2.14-2.40 minutes respectively. Low values of the oil soluble suppositories are attributed to the low melting point of the bases while PEG bases has higher melting point but their water soluble properties made them easy to dissolve. PEG bases suppositories do not melt at body temperature but rather dissolve slowly in the body’s fluids. This property permits the slow release of drug from the suppository and PEG bases suppository show higher disintegration time as compared to oil soluble bases suppositories. Similar findings were also obtained by Suleiman *et al*,^12^ in their study.

After six cycles of freeze-thaw process, when inspected visually, then it was found that water soluble suppositories had no significant sign of instability, while oil soluble suppositories became harder. When they were kept for accelerated stability testing, again water soluble suppositories had no sign of instability while oil soluble suppository became too soft, which is a sign of instability. When drug content was determined in all the suppositories then they were well with in limit.

*In vitro* dissolution studies revealed that release rate was higher in water soluble base compared to the oil soluble bases. This enhancement of the dissolution was due to the solubility of the indomethacin in water-soluble bases. PEG bases act as solid dispersion of the indomethacin. Effect of the surfactant (Tween-80) was also studied on the dissolution rate. Dissolution rate was increased when Tween-80 was used in low concentration (1%), whereas at higher concentration (5%) dissolution rate was not proportionally increased. It was found that at higher concentration surfactant forms micelle, from which drug does not escape easily. So dissolution rate was not as higher as expected.

## References

[1] Borne RF, Williams AD, Lemke LT (2002). Nonsteroidal anti-inflammatory agents. Foye’s principle of medicinal chemistry.

[2] Roberts II LJ, Morrow DJ, Hardman JG, Limbird LE, Gilman AG (2001). Analgesic-antipyretic and anti-inflammatory agents. Goodman and Gilman’s the pharmacological basis of therapeutics.

[3] Katzung BG, Furst DE, Katzung BG (1998). Nonsteroidal anti-inflammatory drugs: Disease-modifying antirheumatic drugs; Nonopoid analgesics; Drugs used in Gout. Basic and clinical pharmacology.

[4] Das KP, Bhattacharya SK, Sen P (1995). Pharmacology. B I Churchill Livingstone.

[5] Tripathi KD (2003). Essentials of medical pharmacology.

[6] Allen LV, Popovich NG, Ansel HC (2005). Ansel’s Pharmaceutical Dosage Forms and Drug Delivery Systems.

[7] Othman S, Muti H (1986). The effect of bases and formulation on the release of Indomethacin from suppositories. Drug Develop Ind Pharm.

[8] Carter SJ (1987). Cooper and Gunn’s dispensing for pharmaceutical students.

[9] (1996). The Indian Pharmacopoeia.

[10] Krowczynsky L (1970). Suppositories in New Medicinal Practice.

[11] Razack MA (2003). Thesis. M. Pharm (IP). SGSITS Indore.

[12] Suleiman MS, Najib NM (1990). Release of indomethacin from suppository bases. Drug Develop Ind Pharm.

